# Urine Culture in Hospitalized Patients during 2014-2018: An Analysis on Pathogen Distribution and Drug Sensitivity

**DOI:** 10.1155/2021/6646024

**Published:** 2021-09-15

**Authors:** Dongkai Sun, Peishan Cong, Fengju Guan, Shuai Liu, Lijiang Sun, Guiming Zhang

**Affiliations:** ^1^Department of Urology, The Affiliated Hospital of Qingdao University, Qingdao 266003, China; ^2^Department of Clinical Laboratory, The Affiliated Hospital of Qingdao University, Qingdao 266003, China

## Abstract

**Objective:**

We sought to analyze the distribution and antibiotic sensitivity of pathogens in hospitalized patients and to provide a scientific reference for the rational application of antibiotics.

**Methods:**

From January 2014 to December 2018, urine cultures from patients in our hospital were collected and analyzed retrospectively for the presence, distribution, and drug sensitivity of pathogens.

**Results:**

A total of 42,854 midstream urine cultures were collected from which 11,891 (27.75%) pathogens were isolated, including 8101 (68.13%) strains of gram-negative bacteria, 2580 (21.69%) strains of gram-positive bacteria, and 1210 (10.18%) strains of fungi. *Escherichia coli* and *Enterococci* were the most common species of gram-negative and gram-positive bacteria, respectively. Drug sensitivity varied among different pathogens. Clear drug resistance was observed in bacteria, while fungus exhibited relatively lower resistance.

**Conclusion:**

Pathogens responsible for urinary tract infections in hospitalized patients are diversiform and display resistance to some antibiotics. Drug resistance monitoring should be enhanced to optimize antimicrobial therapy.

## 1. Introduction

Urinary tract infections (UTIs) are well established as common clinical infections, caused mainly by gram-negative bacteria. UTI diagnosis is based largely on clinical symptoms, with the support of nonspecific laboratory tests, such as urinalysis and urine culture [1, 2]. UTIs are classified as either lower (confined to the bladder) or upper (pyelonephritis) and as uncomplicated or complicated [3]. The annual incidence of physician-diagnosed UTIs in the United States is greater than 10% for females and 3% for males, and more than 60% of females will be diagnosed with a UTI in their lifetime [4]. UTIs not only have a high incidence rate but also negatively impact people's health, including quality of life and medical costs. At present, patients with UTIs are often first treated with empirical antimicrobial therapy. The widespread use of antibiotics, however, imposes strong selective pressure for the development of antibiotic resistance. In addition, in recent years, with the development of new broad-spectrum antibiotics and their clinical application, drug resistance in pathogens causing UTIs has become increasingly serious. In a study by Arana and colleagues on the prevalence and evolution of multidrug resistance (MDR) profiles, the proportion of hospitalized patients with UTIs caused by *Escherichia coli* (*E. coli*) resistant to amoxicillin and ciprofloxacin increased from 5.89% in 2007–2010 to 8.18% in 2011–2014 [5]. Additionally, *Klebsiella pneumoniae* drug resistance is becoming increasingly common. Guidelines by the European Association of Urology (2019 edition) suggest that short-range fluoroquinolones may be used as first-line treatment for patients with uncomplicated pyelonephritis. However, data from the China Antimicrobial Surveillance Network (2019 edition) indicates that only 38.2% of *E. coli* strains were sensitive to ciprofloxacin, decreasing by 4% compared with the past year. In addition, a European survey on mortality caused by drug-resistant bacterial infections reported that carbapenem-resistant *K. pneumoniae* and *E. coli* were the main pathogens causing the fastest increase in patient mortality [6]. Previous studies on the drug resistance of pathogenic bacteria from urine samples were limited by small sample sizes and relatively short study periods, as well as regional discrepancies. In the present study, we aimed to explore the distribution and drug resistance of pathogenic bacteria in urine cultures of inpatients in a regional central hospital during the past 5 years.

## 2. Materials and Methods

### 2.1. Sample Collection

We conducted a retrospective, consecutive study in a regional central hospital in eastern China. A total of 42,854 urine culture specimens from January 2014 to December 2018 were collected. If multiple urine culture results of a patient during hospitalization showed the same bacteria, only one result was included. Informed consent was signed for each participant. The study was approved by the Ethics Review Committee of the Affiliated Hospital of Qingdao University.

### 2.2. Strain Identification and Antibiotic Susceptibility Tests

Isolates were identified by matrix-assisted laser desorption ionization-time of flight mass spectrometry (MALDI-TOF MS; Bruker, Germany). Susceptibility tests were performed using the VITEK 2 automated system (bioMérieux, France) supplemented with the Kirby Bauer disk diffusion method according to the recommendations of the Clinical and Laboratory Standards Institute (CLSI). *E. coli* ATCC 25922, *Staphylococcus aureus* ATCC 25923, and *Pseudomonas aeruginosa* ATCC 27853 were used as quality control strains.

### 2.3. Statistical Analysis

SPSS 24.0 software was used for statistical analysis. The categorical variables were analyzed using Pearson's chi-square test. *P* values ≤ 0.05 were considered statistically significant.

## 3. Results

### 3.1. Pathogen Distribution of Urine Cultures

A total of 42,854 urine culture specimens were examined from which 11,891 strains of pathogens were isolated, yielding a positive rate of 27.75%. Among them, 8101 (68.13%) were gram-negative bacteria, 2580 (21.69%) were gram-positive bacteria, and 1210 (10.18%) were strains of fungi.

*E. coli* (4845 strains), *K. pneumoniae* (972 strains), *P. aeruginosa* (422 strains), *Proteus singularis* (390 strains), and *Acinetobacter baumannii* (142 strains) were the five most prevalent strains of gram-negative bacteria. The most common gram-positive strains were *Enterococcus faecalis* (1176 strains), *Enterococcus faecium* (731 strains), *Staphylococcus epidermidis* (151 strains), and *S. aureus* (70 strains). *Candida albicans* (480 strains) and *Candida tropicalis* (246 strains) were the most common fungi (Table 1).

### 3.2. Extended-Spectrum *β*-Lactamase (ESBL) Detection

A total of 4845 *Enterobacteriaceae*-positive urine cultures were identified from the clinical laboratory in this hospital. ESBL-*E. coli* was found in 2164 samples, with a corresponding overall prevalence of 53.25%. The ESBL producing rate of *K. pneumoniae* was 42.78%.

### 3.3. Drug Resistance of Main Pathogens to Common Antimicrobials

#### 3.3.1. *E. coli* Resistance Rate to Common Antibiotics

The drug sensitivity of *E. coli* is shown in Table 2. The antibacterial drugs with the highest efficacy against *E. coli* were imipenem, meropenem, amikacin, furantoin, piperacillin/tazobactam, and cefoperazone/sulbactam.

#### 3.3.2. Common Antibiotic Sensitivity of *K. pneumoniae*

As shown in Table 3, *K. pneumoniae* was highly sensitive to amikacin, cefoperazone/sulbactam, piperacillin/tazobactam, imipenem, and meropenem. However, imipenem- and meropenem-positive *K. pneumoniae* strains were found in urine cultures from 2015.

#### 3.3.3. Sensitivity of the Two *Enterococci* to Common Antibiotics

The sensitivity of the two *Enterococci* species to common antibiotics is shown in Tables 4 and 5. *E. faecium* had low sensitivity to penicillin G, ampicillin, erythromycin, and ciprofloxacin. No linezolid-, teicoplanin-, and tigecycline-resistant strains were detected. While strains displayed generally high sensitivity to vancomycin, a small number of drug-resistant strains appeared. *E. faecalis* was more sensitive to penicillin G, ampicillin, and nitrofurantoin, and no strains resistant to teicoplanin and tigecycline were detected. However, a few strains resistant to vancomycin and linezolid were cultured.

#### 3.3.4. Fungal Drug Resistance

The overall drug resistance rate of *C. tropicalis*, *C. albicans*, and *Candida smooth* was low; only a few *C. tropicalis* strains resistant to fluconazole and itraconazole were found. Moreover, the drug resistance rate of *C. smooth* to itraconazole was 14.64%.

## 4. Discussion

In recent years, UTI-associated pathogens and their susceptibility patterns have changed significantly worldwide [7]. The abuse of antimicrobial agents is an important factor in the development of complex UTIs. As a result, although the types of antibacterial drugs are constantly being updated, drug resistance is also increasing and is especially true for patients with mixed infections of multiple pathogenic bacteria. The main risks associated with the overuse of antibiotics are the induction of drug resistance, increased possibility of pathogenic strains, and the occurrence of double infections [8]. Understanding the distribution of bacterial infections in Chinese hospitals will be crucial for the development of treatment guidelines designed to reduce hospital-acquired infections and drug resistance. In this study, we characterized the pathogenic infections of a large sample of 42,854 urine cultures from the hospital of Qingdao University of Medicine from 2014 to 2018.

Within the 5-year period of this study, a few significant trends in pathogen distribution were observed, and our findings were roughly consistent with studies conducted at other hospitals in China and abroad. We found that the main pathogenic agents responsible for causing UTIs in hospitalized patients were gram-negative bacteria (68.13%), gram-positive bacteria (21.69%), and fungi (10.18%), among which *E. coli* was the dominant bacteria, which is roughly consistent with reports by Raka et al. [9]. ESBL-producing *E. coli* and *K. pneumoniae* detection rates were 53.25% and 42.78%, respectively. *E. coli* has a high rate of drug resistance to a variety of common cephalosporins, which is speculated to be mainly because of the overuse of these drugs (especially third-generation variants) in recent years. This had led to the emergence and spread of ESBL strains of *E. coli* and an increase in drug-resistant strains because of the conversion of sensitive bacteria into drug-resistant strains [10]. According to literature reports, for several classes of antimicrobial agents in clinical use, such as fluoroquinolones and cephalosporins (second and third generation), the resistance rates of ESBL strains were over 50%. As such, it is now recommended that these drugs should not be used as the treatments of choice for complex cystitis, but instead fosfomycin and nitrofurantoin are recommended. In addition to carbapenems, cefoperazone/sulbactam, and piperacillin/tazobactam, several new drugs have been developed and applied for treatment of ESBL-producing gram-negative bacterial infections in recent years. Foreign clinical trials have reported that ceftazidime combined with avibatan has good efficacy against ESBL-producing gram-negative bacteria [11]. Ceftolozane/tazobactam, a new antibacterial drug, also showed strong antibacterial activity against MDR and ESBL-producing *E. coli* in complex UTI treatment [12]. Although these drugs have good efficacy, they are costly and require intravenous administration, causing a burden to certain patients.

Our results showed that the drug resistance rate of *E. coli* to common fluoroquinolones (ciprofloxacin and levofloxacin) was more than 60.00%, and Yang et al. reported that the sensitivity rate of UTI-related *E. coli* to these two drugs was only 30% [13]. This seems to suggest that levofloxacin is no longer suitable for initial empirical treatment of UTIs. However, clinical studies have found that levofloxacin has good efficacy in the treatment of UTIs, with a clearance rate of over 90% for common pathogenic bacteria such as *E. coli* [14]. Levofloxacin is still recommended in European guidelines as a first-line treatment for pyelonephritis, and domestic guidelines recommend it as a first-line treatment for initial empirical treatment of uncomplicated UTIs. With high bioavailability, the concentration of levofloxacin in urine within 24 h is far higher than the 90% bacteriostatic and bactericidal concentration of common pathogenic bacteria. Therefore, whether the high drug resistance rate in the laboratory can be used as an indicator of empirical drug use still needs further in-depth research. In addition, compared with intravenous administration, oral cephalosporins have a low concentration in urine and may not be able to achieve effective bactericidal concentrations. Therefore, in clinical drug selection, the pharmacokinetics of drugs should be considered as well as drug sensitivity results to avoid inappropriate selection and to choose the most appropriate drug and drug regimen to improve UTI treatment efficiency.

*E. coli* and *K. pneumoniae* had low resistance rates to carbapenem antibacterial drugs, which is consistent with the report by Nozarian and Abdollahi [15]. However, the number of *E. coli* strains resistant to carbapenems has been on the rise in recent years. As carbapenem-resistant strains are often resistant to other common antimicrobial drugs, situations in which no effective drugs are available for treatment can occur, which in turn leads to a high mortality rate because of infection [16]. In general, enzymatic higher antimicrobial resistance mainly imine culture south of penicillium carbon alkene antimicrobial drug, but at the moment because of such widespread use or even abuse, antimicrobial drugs in clinical pathogenic bacteria resistance, great changes have taken place, and then, part of it has been reported at home and abroad of gram-negative bacteria of penicillium carbon alkene resistance phenomenon and showed a trend of diffusion [17]. Because of its high enzymatic resistance, clinical attention should be paid to drug resistance of this kind of drug.

In this study, *E. faecalis* and *E. faecium* ranked second and fourth in the distribution of pathogenic bacteria in the urine cultures, respectively, accounting for 16.04% of pathogens detected. The high proportion of *Enterococci* is related to the complicated factors of the urinary tract, the use of immunosuppressive agents, the increase of invasive procedures in hospitals, and the unreasonable use of antibiotics in the elderly population. *Enterococci* are one of the main causes of nosocomial infections, and because of their inherent resistance to many kinds of antimicrobial agents and acquired drug resistance, fewer classes of drugs are available to treat these infections. In this study, *Enterococci* showed almost no resistance to linezolid, vancomycin, teicoplanin, and tigecycline. However, these drugs are expensive and should be used sparingly. Furthermore, *E. faecalis* and *E. faecium* show significant differences in drug resistance. Therefore, in the treatment of enterococcal infections, corresponding antimicrobial agents should be selected according to the interspecific differences in drug resistance. *E. faecalis* is highly sensitive to furantoin and, because of its low price, is often used as the drug of choice. *Enterococci* are almost 100.0% sensitive to glycopeptides and linezolid, and the concentration of glycopeptides in urine is relatively high, which suggests that glycopeptides are still the preferred antimicrobial agents for the treatment of enterococcal-induced UTIs, including severe MRSA infections [18]. The principle of “deescalation” was adopted in antibiotic administration, stressing “early use, early stop” and “perioperative application.” Antibiotics were applied to wounds or administered systemically when they could not be applied to burn wounds. Similar strategies have been followed in several guidelines [19–21].

Fungal UTIs are usually rare, but *C. albicans* may rise to become the most prevalent pathogenic agent in patients with long-term indwelling catheters [22]. In this study, a total of 1210 strains of fungi were detected, accounting for 10.18% of the total number of pathogens detected, among which *C. albicans* and *C. tropicalis* accounted for 4.04% and 2.07%, respectively. We identified a higher rate of fungal pathogens in 2017 than the other four years. These isolates were highly sensitive to fluconazole and voriconazole, with a resistance rate under 10%, while resistance to itraconazole was more prevalent. Some risk factors have been elucidated for fungal infection in urinary tract, such as diabetes mellitus, pregnancy, recent antibiotic usage or surgical procedures, urinary tract instrumentation and indwelling catheters, urinary tract disease (including neurogenic bladder, urolithiasis, and bladder outlet obstruction), immunosuppressive medication usage, and renal transplantation [23]. Further investigation will be required to determine the impact of patient age, antibiotic administration, sedentary lifestyle during hospitalization, and long-term catheterization on these high rates of infection.

## 5. Conclusions

*E. coli*, *Enterococcus*, and *K. pneumoniae* are common causes of UTIs in hospitalized patients, and their resistance to common antibacterial drugs is a serious issue. Drug-resistant bacteria often lead to longer hospital stays and higher treatment costs [24]. Therefore, clinicians should combine the characteristics of specific drugs in urine culture with drug sensitivity tests and detection results of special drug-resistant strains to rationally use antibacterial drugs and to achieve the ideal therapeutic effect. Furthermore, blind empirical use should be avoided to reduce or control the variation and drug resistance rate of pathogenic bacteria.

## Figures and Tables

**Table 1:** Distribution and proportion of pathogenic bacteria in urine culture from 2014 to 2018.

Pathogen	Strain (*n*)	Percentage (%)
*G−*	8101	68.13
*Escherichia coli*	4845	40.76
*Klebsiella pneumoniae*	972	8.17
*Pseudomonas aeruginosa*	422	3.55
*Proteus mirabilis*	390	3.28
*Acinetobacter baumannii*	142	1.19
Other	1330	11.18
*G+*	2580	21.69
*Enterococcus faecium*	1176	9.89
*Enterococcus faecalis*	731	6.15
*Staphylococcus epidermidis*	151	1.27
*Staphylococcus aureus*	70	0.59
Other	451	3.79
*Fungus*	1210	10.18
*Candida albicans*	480	4.04
*Candida tropicalis*	246	2.07
*Candida glabrata*	271	2.28
Other	484	4.07
Total	11891	100

**Table 2:** Antimicrobial resistance of *Escherichia coli* in urine culture from 2014 to 2018.

	2014		2015		2016		2017		2018	
	Strain (*n*)	Percentage (*χ*/%)	Strain (*n*)	Percentage (*χ*/%)	Strain (*n*)	Percentage (*χ*/%)	Strain (*n*)	Percentage (*χ*/%)	Strain (*n*)	Percentage (*χ*/%)
Amikacin	43	4.86	32	3.37	8	2.68	31	2.99	20	1.88
Amoxicillin	83	95.40	5	83.33	—	—	—	—	—	—
Amoxicillin/clavulanic acid	67	12.41	99	17.97	27	9.06	50	8.77	46	13.81
Cefoperazone/sulbactam	10	3.07	28	4.33	9	3.02	55	7.05	56	6.78
Cefazolin	502	63.14	576	70.50	171	57.77	626	65.76	591	56.39
Cefoxitin	128	23.70	138	25.09	56	18.79	90	15.85	59	17.72
Cefepime	369	44.67	383	40.57	60	20.13	197	18.92	169	15.87
Cefuroxime	20	71.43	1	100.00	—	—	79	64.23	390	53.29
Levofloxacin	547	68.63	657	69.67	179	60.07	666	63.98	672	63.10
Ciprofloxacin	637	71.98	682	71.94	186	62.42	702	67.50	690	64.79
Moxifloxacin	269	69.87	252	71.39	79	61.24	—	—	—	—
Ceftazidime	257	59.49	213	52.72	—	—	140	22.99	220	20.81
Ceftriaxone	468	58.72	530	56.26	157	52.68	568	54.62	544	51.13
Meropenem	12	1.88	11	1.28	3	1.01	7	0.7	17	1.69
Aztreonam	413	51.82	448	47.71	106	35.57	393	37.82	334	31.42
Nitrofurantoin	45	5.26	56	5.93	8	2.82	38	3.69	50	4.76
Gentamicin	484	54.69	446	47.10	148	49.66	469	45.1	452	42.56
Compound sulfamethoxazole	589	66.55	556	58.65	175	58.72	577	55.43	573	53.85
Imipenem	10	1.13	14	1.48	3	1.01	7	0.67	20	1.88
Piperacillin/tazobactam	29	3.51	21	2.23	7	2.35	17	1.63	27	2.54

**Table 3:** The antimicrobial resistance rate of *Klebsiella pneumoniae* in urine culture from 2014 to 2018.

	2014		2015		2016		2017		2018	
	Strain (*n*)	Percentage (%)	Strain (*n*)	Percentage (%)	Strain (*n*)	Percentage (%)	Strain (*n*)	Percentage (%)	Strain (*n*)	Percentage (%)
Amikacin	8	4.08	3	1.55	3	4.84	14	6.48	8	3.43
Amoxicillin	14	100.00	—	—	—	—	—	—	—	—
Amoxicillin/clavulanic acid	21	19.44	27	24.11	11	17.74	31	21.23	16	23.88
Cefoperazone/sulbactam	1	1.43	10	7.52	9	14.52	25	14.37	17	9.29
Cefazolin	111	60.99	97	60.62	45	72.58	116	57.43	100	43.29
Cefoxitin	20	18.52	24	21.43	11	17.74	31	21.23	12	17.91
Cefepime	76	40.43	57	29.38	10	16.13	36	16.67	24	10.30
Cefuroxime	2	33.33	1	100.00	—	—	—	—	45	41.28
Levofloxacin	60	32.97	62	32.29	25	40.32	53	24.54	46	19.74
Ciprofloxacin	80	40.82	78	40.21	26	41.94	60	27078	60	25.75
Moxifloxacin	32	42.67	34	44.16	8	36.36	—	—	—	—
Ceftazidime	51	50	31	37.8	—	—	—	—	35	20.71
Ceftriaxone	98	53.85	87	44.85	43	69.35	96	44.44	81	34.76
Meropenem	0	0	7	3.93	6	9.68	7	3.32	4	1.82
Aztreonam	87	47.8	77	39.9	22	35.48%	71	32.87	52	22.41
Nitrofurantoin	101	53.16	72	37.7	21	38.89	76	36.54	83	36.24
Gentamicin	69	35.20	47	24.23	22	35.48	67	31.02	55	23.61
Compound sulfamethoxazole	96	48.98	105	54.12	35	56.45	106	49.07	97	41.63
Imipenem	0	0.00	5	2.59	6	9.68	7	3.26	7	3.00
Piperacillin/tazobactam	8	4.28	13	6.70	8	12.90	12	5.56	11	4.72

**Table 4:** Total drug resistance rate of *Enterococcus faecium* cultured in urine from 2014 to 2018.

	2014		2015		2016		2017		2018	
	Strain (*n*)	Percentage (%)	Strain (*n*)	Percentage (%)	Strain (*n*)	Percentage (%)	Strain (*n*)	Percentage (%)	Strain (*n*)	Percentage (%)
Penicillin G	163	97.02	214	96.83	100	99.01	82	98.80	218	98.20
Ampicillin	168	96.55	218	96.89	100	97.09	301	96.47	218	98.20
Ciprofloxacin	161	92.53	212	94.22	102	99.03	284	91.03	199	89.64
Nitrofurantoin	81	46.55	144	64.00	69	69.00	179	57.74	115	55.29
Erythromycin	160	91.95	211	93.78	90	87.38	283	90.71	200	90.09
Tetracycline	73	41.95	118	52.44	56	54.37	191	61.22	123	55.41
Linezolid	0	0.00	0	0.00	0	0.00	0	0.00	0	0.00
Vancomycin	4	2.30	0	0.00	0	0.00	2	0.64	2	0.90
Teicoplanin	0	0.00	0	0.00	0	0.00	0	0.00	0	0.00
Tigecycline	0	0.00	0	0.00	0	0.00	0	0.00	0	0.00
Gentamicin	133	76.44	158	70.22	62	60.78	175	56.27	117	52.70

**Table 5:** Total drug resistance rate of *Enterococcus faecalis* cultured in urine from 2014 to 2018.

	2014		2015		2016		2017		2018	
	Strain (*n*)	Percentage (%)	Strain (*n*)	Percentage (%)	Strain (*n*)	Percentage (%)	Strain (*n*)	Percentage (%)	Strain (*n*)	Percentage (%)
Penicillin G	13	11.61	51	29.65	5	11.11	2	5.13	7	4.49
Ampicillin	13	10.83	53	29.94	4	8.89	5	2.94	5	3.21
Ciprofloxacin	48	40.00	57	32.20	14	31.11	65	38.24	52	33.33
Nitrofurantoin	2	1.67	1	0.57	1	2.22	1	0.59	6	3.90
Erythromycin	92	76.67	132	74.58	36	80.00	117	68.82	112	71.79
Tetracycline	106	88.33	156	88.14	38	84.44	146	85.88	137	87.82
Linezolid	2	1.85	2	1.27	1	2.44	2	1.23	0	0
Vancomycin	1	0.83	0	0.00	1	2.22	1	0.59	1	0.64
Teicoplanin	0	0.00	0	0.00	0	0.00	0	0.00	0	0.00
Tigecycline	0	0.00	0	0.00	0	0.00	0	0.00	0	0.00
Gentamicin	54	45.00	89	50.86	23	52.27	81	47.93	71	45.51

## Data Availability

The data used to support the findings of this study are included within the article.

## References

[1] Grigoryan L., Trautner B. W., Gupta K. (2014). Diagnosis and management of urinary tract infections in the outpatient Setting. *BMC Musculoskeletal Disorders*.

[2] Chu C., Lowder J. L. (2018). Diagnosis and treatment of urinary tract infections across age groups. *American journal of obstetrics and gynecology*.

[3] Flores-Mireles A., Walker J. N., Caparon M., Hultgren S. J. (2015). Urinary tract infections: epidemiology, mechanisms of infection and treatment options. *Nature reviews microbiology*.

[4] Klein R. D., Hultgren S. J. (2020). Urinary tract infections: microbial pathogenesis, host-pathogen interactions and new treatment strategies. *Nature Reviews Microbiology*.

[5] Arana D. M., Rubio M., Alós J. I. (2017). Evolucion de la multirresistencia a los antibioticos en *Escherichia coli y Klebsiella pneumoniae* aislados de infecciones del tracto urinario. Un an alisis de 12 años (2003-2014). *Enfermedades Infecciosas y Microbiología Clínica*.

[6] Cassini A., Högberg L., Plachouras D. (2019). Attributable deaths and disability-adjusted life-years caused by infections with antibiotic-resistant bacteria in the EU and the European Economic Area in 2015: a population-level modelling analysis. *The Lancet infectious diseases*.

[7] Gilani S. Y., Shah S. R., Ahmad N., Bibi S. (2016). Antimicrobial resistance patterns in community acquired urinary tract infections. *Journal of Ayub Medical College Abbottabad*.

[8] Singh R., Rohilla R., Sangwan K., Siwach R., Magu N. K., Sangwan S. S. (2011). Bladder management methods and urological complications in spinal cord injury patients. *Indian journal of orthopaedics*.

[9] Raka L., Mulliqi-Osmani G., Berisha L. (2004). Etiology and susceptibility of urinary tract isolates in Kosova. *International journal of antimicrobial agents.*.

[10] Marijan T., Plecko V., Vranes J., Dzepina A. M., Bedenić B., Kalenić S. (2010). Characterization of ESBL-producing *Escherichia coli* and *Klebsiella pneumoniae* strains isolated from urine of nonhospitalized patients in the Zagreb region. *Medicinski Glasnik*.

[11] Livermore D. M., Mushtaq S. (2013). Activity of biapenem (RPX2003) combined with the boronate -lactamase inhibitor RPX7009 against carbapenem-resistant *Enterobacteriaceae*. *Journal of Antimicrobial Chemotherapy*.

[12] Sader H. S., Farrell D. J., Flamm R. K., Jones R. N. (2014). Ceftolozane/tazobactam activity tested against aerobic Gram-negative organisms isolated from intra-abdominal and urinary tract infections in European and United States hospitals (2012). *Journal of Infection*.

[13] Yang Q., Zhang H., Wang Y. (2017). Antimicrobial susceptibilities of aerobic and facultative gram-negative bacilli isolated from Chinese patients with urinary tract infections between 2010 and 2014. *BMC infectious diseases*.

[14] Zhang Y., Xu J. F., Wu X. J. (2009). Population pharmacokinetics of oral levofloxacin 500 mg once-daily dosage in community-acquired lower respiratory tract infections: results of a prospective multicenter study in China. *Journal of infection and chemotherapy*.

[15] Nozarian Z., Abdollahi A. (2015). Microbial etiology and antimicrobial susceptibility of bactria implicated in urinary tract infection in Tehran, Iran. *Iranian Journal of Pathology*.

[16] Hu F., Guo Y., Zhu D. (2016). Resistance trends among clinical isolates in China reported from CHINET surveillance of bacterial resistance, 2005-2014. *Clinical microbiology and infection*.

[17] Nordmann P., Cuzon G., Naas T. (2009). The real threat of *Klebsiella pneumoniae* carbapenemase-producing bacteria. *The Lancet infectious diseases*.

[18] Bubacz M. R. (2007). Community-acquired Methicillin-Resistant *Staphylococcus Aureus* an ever-emerging epidemic. *Aaohn Journal*.

[19] Avni T., Levcovich A., Ad-el D. D., Leibovici L., Paul M. (2010). Prophylactic antibiotics for burns patients: systematic review and meta-analysis. *BMJ*.

[20] Hooton T., Bradley S., Cardenas D. (2010). Diagnosis, prevention, and treatment of catheter-associated urinary tract infection in adults: 2009 International Clinical Practice Guidelines from the Infectious Diseases Society of America. *Clinical infectious diseases*.

[21] Sirijatuphat R., Pongsuttiyakorn S., Supapueng O., Kiratisin P., Thamlikitkul V. (2020). Implementation of global antimicrobial resistance surveillance system (GLASS) in patients with bacteriuria. *Journal of global antimicrobial resistance*.

[22] Timurkaynak F., Arslan H., Kurt Azap Ö. (2008). In vitro activity of tigecycline against resistant micro-organisms isolated from burn patients. *Burns*.

[23] Kauffman C. A., Vazquez J. A., Sobel J. D. (2000). Prospective multicenter surveillance study of funguria in hospitalized Patients. *Clinical Infectious Diseases*.

[24] Ubbink D. T., Santema T. B., Stoekenbroek R. M. (2014). Systemic wound care: a meta-review of Cochrane systematic reviews. *Surgical Technology International*.

